# Imbalance of Autophagy and Apoptosis Induced by Oxidative Stress May Be Involved in Thyroid Damage Caused by Sleep Deprivation in Rats

**DOI:** 10.1155/2021/5645090

**Published:** 2021-09-10

**Authors:** Yongmei Li, Wei Zhang, Mengqi Liu, Qiuping Zhang, Zijie Lin, Miao Jia, Dan Liu, Laixiang Lin

**Affiliations:** ^1^NHC Key Laboratory of Hormones and Development, Tianjin Key Laboratory of Metabolic Diseases, Chu Hsien-I Memorial Hospital & Tianjin Institute of Endocrinology, Tianjin Medical University, Tianjin, China; ^2^Basic Medical College, Tianjin Medical University, Tianjin, China; ^3^Qinghai Institute for Endemic Disease Prevention and Control, Qinghai, China

## Abstract

Many studies have shown that sleep deprivation can affect a wide range of tissues and organs, and most of these effects are related to oxidative stress. Oxidative stress can cause functional and morphological changes in cells, which are closely related to autophagy and apoptosis. In this study, we examined changes in thyroid morphology and function, oxidative stress, autophagy, and apoptosis in rats after sleep deprivation. Thyroid hormones, thyroid-stimulating hormone, functional substances required for the synthesis of thyroid hormones, and thyroid morphological observations were used to evaluate the changes and impairment of thyroid function. Methane dicarboxylic aldehyde and total antioxidant capacity were measured to assess oxidative stress in the thyroid. To evaluate the balance of autophagy and apoptosis, the expression of autophagy- and apoptosis-related proteins was examined by western blotting, and apoptotic cells were labeled with TUNEL staining. The body weight of rats in the sleep deprivation group decreased, but the relative weight of the thyroid gland increased. Sleep deprivation led to morphological changes in the thyroid. The levels of thyroid hormones and thyroid-stimulating hormone increased after sleep deprivation. Total antioxidant capacity decreased, and methane dicarboxylic aldehyde levels increased in the thyroid in the sleep deprivation group. Analysis of autophagy- and apoptosis-related proteins indicated that the microtubule-associated protein 1 light chain 3 beta- (LC3B-) and LC3A-II/I ratio and Beclin 1 levels significantly decreased in the sleep deprivation group and P62 levels significantly increased. The number of apoptotic cells in the thyroid gland of sleep-deprived rats increased significantly. Taken together, these results indicate that sleep deprivation can lead to oxidative stress in the thyroid and ultimately cause thyroid damage, which may be related to the imbalance of autophagy and apoptosis.

## 1. Introduction

Sleep deprivation is the condition of having an insufficient amount or quality of sleep due to circadian rhythm disturbance, the influence of the external environment, or various other factors. With increased pressure from work and life in modern society, sleep deprivation has become a widespread and serious threat to human health. In recent years, many researchers have investigated the damage caused to the body by sleep deprivation, including to the nervous system, digestive system, and memory [1, 2]. Sleep deprivation is generally thought to lead to an imbalance between the body's oxidant and antioxidant systems, resulting in increased levels of oxygen free radicals, which damage the membrane system of cells, ultimately causing abnormal organ morphology and function [3].

Autophagy is a normal metabolic process but can also trigger apoptosis. Many studies have demonstrated that mammals have two signaling pathways that regulate autophagy, namely, the Bcl-2-Beclin 1 signaling pathway [4] and the PI3K-mTOR signaling pathway [5]. The former activates autophagy, whereas the latter inhibits it. Upon initiation of autophagy, cytoplasmic microtubule-associated protein 1 light chain 3 beta (LC3B) changes to the form of the protein found in the autophagosome membrane. The formation of phagosomes is regulated by the autophagy protein Beclin 1, which forms a multiprotein complex with PI3KC3 and other proteins, thereby activating the second messenger PI3P to promote the formation of autophagosomes. Beclin 1, LC3, and P62 are the three most important proteins in the process of autophagy. Autophagy is activated by Beclin 1, and then, P62 binds to LC3-II and is degraded. As a result, P62 is consumed and its expression is decreased. Therefore, measurement of these three proteins can directly reflect the degree of autophagy in animal cells.

The Bcl-2 gene family, which is composed mainly of *Bcl-2*, *Bax*, *Bak*, *Mcl-1*, and *Bcl-x*, is an important contributor to the regulation of apoptosis. *Bcl-2* is a protooncogene, whereas *Bax* encodes Bcl-2-associated protein X. The Bax monomer cannot induce apoptosis, but when Bax and Bcl-2 combine to form a dimer, Bcl-2 is inactivated, thereby promoting apoptosis; Bax can also form dimers with itself, which directly promote apoptosis. Correlation analysis between apoptosis and gene expression has shown that the Bax/Bcl-2 ratio has a significant positive correlation with apoptosis in the thyroid. The Bcl-2 protein not only has an antiapoptotic function but also mediates autophagy by interacting with other proteins, suggesting that, in addition to promoting cell death, it can also help to maintain cell survival.

Apoptotic signaling pathways are divided into extracellular and intracellular pathways. The extracellular apoptotic signal is transduced by the death receptor, and its ligands include the tumor necrosis factor [6]. Intracellular stimuli that induce apoptosis include oxidative stress, DNA damage, hypoxia, and loss of growth factors [7].

A few studies have reported that sleep deprivation can cause thyroid damage [8, 9]. Although the reported data indicate that the thyroid is affected by sleep deprivation, the underlying molecular mechanisms have not been studied in depth. In this study, we examined the effects of sleep deprivation on thyroid physiological and biochemical indexes in rats. We also explored the changes and possible roles of autophagy and apoptosis in thyroid injury induced by sleep deprivation.

## 2. Materials and Methods

### 2.1. Animal Source and Grouping

Forty-two healthy male SPF Wistar rats weighing 300–350 g were purchased from the Experimental Animal Center of the PLA Academy of Military Medical Sciences. After acclimatization for 1 week, the rats were divided randomly into a cage control (CC) group, tank control (TC) group, and sleep deprivation (SD) group (each *n* = 14). The experiments were approved by the Animal Ethical and Welfare Committee of Tianjin Medical University Chu Hsien-I Memorial Hospital (Approval No. DXBYY-IACUC-2020001) in accordance with the National Institutes of Health guidelines.

### 2.2. Reagents

Methane dicarboxylic aldehyde (MDA) and total antioxidant capacity (T-AOC) test kits were purchased from the Nanjing Jiancheng Bioengineering Institute (China). The following primary antibodies were used: *β*-actin (ABclonal Biotechnology, Wuhan, China); LC3B (Sigma-Aldrich, St. Louis, MO); P62 (Abcam, Cambridge, MA); and LC3A, Beclin 1, Bcl-2, and Bax (Proteintech Technology, Wuhan, China). A protein assay kit (Biomed, Beijing, China), secondary antibody (Proteintech Technology), polyvinylidene difluoride membranes (0.22 *μ*m pore size), and Immobilon Western Chemiluminescent HRP Substrate (Millipore, Burlington, MA) were used for western blotting. A TUNEL assay kit was used to detect apoptotic cells (Wanleibio, Shenyang, China).

### 2.3. Experimental Design

#### 2.3.1. Sleep Deprivation Procedure

The rats in the CC group engaged in normal activity and rest in their home cages with a 12 h light/dark cycle. Room temperature was controlled at 18.0°C–22.0°C. The rats in the SD group were subjected to sleep deprivation according to a slightly modified version of the “platform on the water” method [10]. A small circular platform (diameter, 63 mm; height, 80 mm) was positioned at the center of a transparent water tank (30 × 30 × 30 cm), which was filled with water (to 2 cm below the level of the platform). The rats were placed on separate platforms for sleep deprivation. Specifically, the rats would awaken after falling into the water because of muscle tone loss during sleep. Each day, the rats were subjected to sleep deprivation for 18 h and allowed to sleep for 6 h. To control the nonspecific effects of environmental factors, the rats in the TC group were placed in an identical tank equipped with a wide platform (diameter, 160 mm) that enabled them to move and sleep freely in a similar water environment as the rats in the SD group. The animals in the TC group were otherwise treated the same as the animals in the CC group. This experiment lasted for 21 days, as in our previous study [11]. Dietary intake was monitored on the day before the end of the experiment. All rats were weighed every two days throughout the study duration.

#### 2.3.2. Biological Sample Collection

On the 21st day, the rats were anesthetized after fasting for 8 h. Blood was collected from the femoral artery. Thyroid tissue was collected for histological and biochemical examinations.

#### 2.3.3. Serum Biochemical and Immunoassay Analysis

Serum samples were collected by centrifugation at 3,000 × *g* for 10 min. A Dxl 800 automatic immunoassay analyzer (Beckman Coulter, Brea, CA) was used to detect thyroid-stimulating hormone (TSH), total T_3_ (TT_3_), total T_4_ (TT_4_), free T_3_ (FT_3_), free T_4_ (FT_4_), and thyroglobulin (TG) antibody in serum according to the appropriate kit protocol.

#### 2.3.4. Detection of Oxidative Stress and Antioxidant Capacity

MDA levels and T-AOC were determined by biochemical methods according to the appropriate kit protocol.

#### 2.3.5. Histological Assessment of the Thyroid

Fresh thyroid tissue was fixed in 10% phosphate-buffered saline-buffered formalin for 48 h and embedded in paraffin. Sections (4 *μ*m thick) were stained with hematoxylin and eosin (HE) and TUNEL. Thyroid tissue morphology was photographed and analyzed under a light microscope (Olympus, Tokyo, Japan). The volume of thyroid follicles was measured and analyzed. The apoptosis index (TUNEL-positive cells of total cells, %) was counted and analyzed. In the above detections, histopathological sections were prepared for 5 rats in each group. Four photos of each section were taken for the measurement, and the average value was obtained.

#### 2.3.6. Western Blotting

Western blotting was used to detect the expression levels of the autophagy-related proteins LC3A, LC3B, and Beclin 1 and the apoptosis-related proteins Bax and Bcl-2 in the rat thyroid gland. Thyroid tissue was cut into pieces and homogenized in RIPA buffer. The tissue homogenate was incubated on ice for 20 min and centrifuged at 13,000 × *g* for 10 min at 4°C. The supernatant was carefully removed, and the protein content in the tissue extract was measured with a BCA protein assay kit (Biomed) according to the manufacturer's instructions. An aliquot of the protein solution was mixed with loading buffer and heated at 100°C for 10 min. The protein solution was separated by sodium dodecyl sulfate-polyacrylamide gel electrophoresis (8%–15%) and transferred to a polyvinylidene difluoride membrane. The membrane was incubated in blocking solution (5% nonfat milk in Tris-buffered saline containing 0.1% Tween-20 (TBST), pH 7.4) for 1 h at room temperature. The membrane was incubated overnight at 4°C with primary antibodies. After washing three times with TBST, the membrane was incubated with secondary antibodies for 2 h at room temperature and washed three times with TBST. The protein bands were visualized with an Immobilon Western Chemiluminescent HRP Substrate (Millipore). Protein abundance was normalized according to *β*-actin levels [11].

### 2.4. Statistical Analysis

The SPSS statistical software package (version 26.0; IBM Corp., Armonk, NY) was used for data analysis. After a Shapiro-Wilk test, normal distributed data were expressed as the mean ± standard deviation (SD) and compared using one-way analysis of variance followed by the least significant difference test (LSD) or Tamhane's multiple comparison test. Abnormal distributed data (or data with less than 6 samples) were presented as the median, compared using the Kruskal-Wallis test, and adjusted by the Bonferroni correction method for multiple tests. A *P* value < 0.05 was adopted as the threshold for significance.

## 3. Results

### 3.1. Effects of Sleep Deprivation on Body and Thyroid Weight in Rats

**Figure 1:**
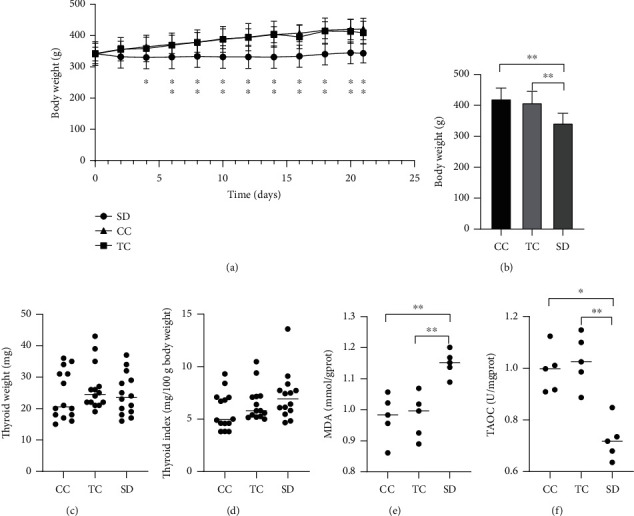
SD affected body weight, thyroid weight, and antioxidant capacity. (a) Body weight changes throughout the study duration (*n* = 14 in each group). (b) Final body weight decreased in the SD group and (c) thyroid weight remained normal in the SD group compared with rats in the CC and TC groups(*n* = 14 in each group). (d) There was a tendency for the thyroid index to increase (*n* = 14 in each group). (e) MDA content increased and (f) T-AOC (total antioxidant capacity) decreased in thyroid tissue compared with rats in the CC and TC groups (*n* = 5 in each group). Data are shown as the mean (bar) and standard deviation (whisker) or median (bar) with scatter plots. ^∗^*P* < 0.05, ^∗∗^*P* < 0.01, compared with rats in the CC or TC group.

As the experiment progressed, the body weight of the rats in each group increased. Weight gain was more obvious in the CC and TC groups. By the end of the experiment, body weight was significantly lower in the SD group compared with the CC and TC groups. There was no obvious difference in the weight of the thyroid gland between the groups. However, the thyroid index tended to increase in the SD group at the end of the experiment compared with the CC and TT groups (Figures 1(a)–1(c)).

### 3.2. Effect of Sleep Deprivation on the Antioxidant Capacity of the Thyroid

Oxidative stress contributes to the mechanism underlying functional changes in the thyroid. Thus, we examined oxidative stress in the thyroid by measuring MDA production and T-AOC. Antioxidant and lipid peroxidation levels were affected by sleep deprivation (Figures 1(e)–1(f)). T-AOC was significantly decreased, and MDA levels were significantly increased in the SD group compared with the CC and TC groups.

### 3.3. Effects of Sleep Deprivation on Thyroid Morphology

**Figure 2:**
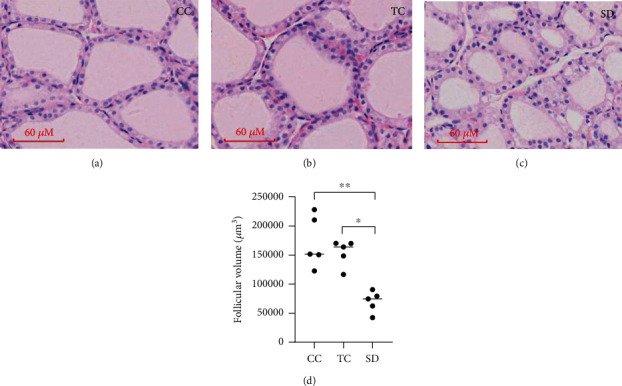
SD affected thyroid morphology. Histopathological changes were found in the rat thyroid on exposure to SD. Representative light microphotographs (magnitude 400x in (a–c) of HE staining of thyroid): (a) thyroid in the CC group; (b) thyroid in the TC group; (c) thyroid in the SD group; (d) follicular volume was measured. Data are shown as the median (bar), and scatter plots are shown. ^∗^*P* < 0.05, ^∗∗^*P* < 0.01, compared with rats in the CC or TC group; *n* = 5 in each group.

Under a light microscope, thyroid cells in the CC group were found to be normal by histopathological analysis. In the SD group, thyroid follicles had normal structure and morphology, but pale cytoplasm and vacuolar degeneration were observed. The volume of some thyroid follicles decreased in the SD group, suggesting the presence of thyroid follicular hyperplasia (Figures 2(a)–2(c)). Follicular volume analysis showed that the volume of thyroid follicles in the SD group was significantly smaller than that in the other two groups.

### 3.4. Effects of Sleep Deprivation on Indicators of Thyroid Function in Rats

**Figure 3:**
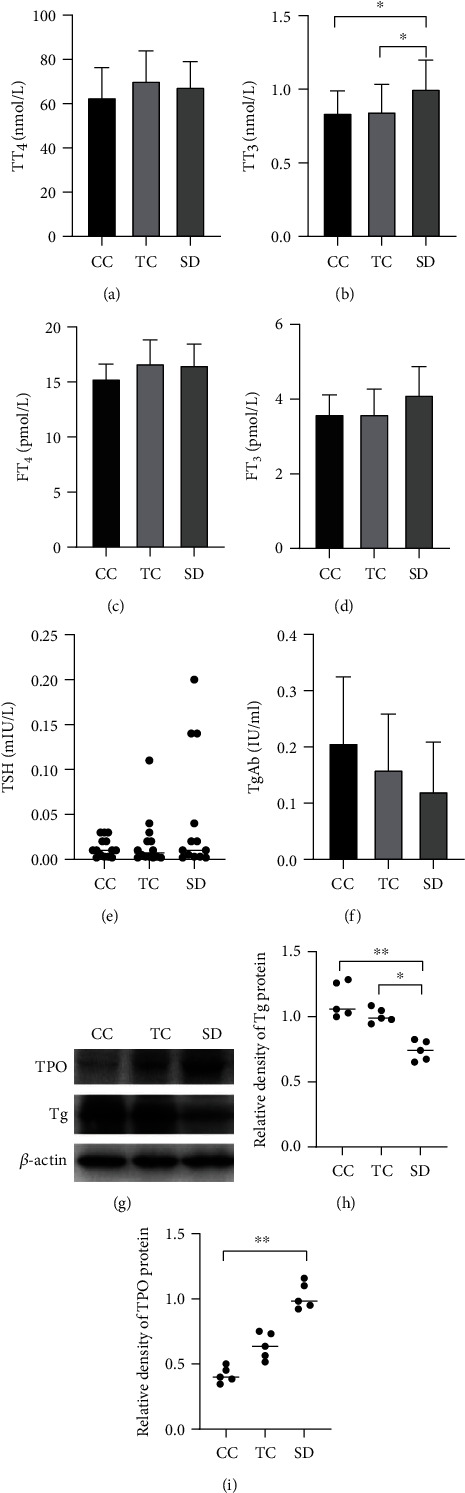
Sleep deprivation induced thyroid function changes in rats. Serum levels of (a) TT4, (b) TT3, (c) FT4, (d) FT3, and (e) TSH were determined. TT3 increased in the SD group compared with the CC and TC groups (*n* = 14 in each group). (g) Western blotting showed that (i) TPO protein expression in the thyroid was higher but (h) Tg protein expression was lower in the SD group compared with the CC group and TC groups (*n* = 5 in each group). Data are shown as the mean (bar) and standard deviation (whisker) or median(bar) with scatter plots showed. ^∗^*P* < 0.05, ^∗∗^*P* < 0.01, compared with rats in the CC or TC group. In TSH comparison, an outlier in the SD group was eliminated.

The serum levels of T_3_ and TSH showed an increasing trend or a significant increase in the SD group (TT_3_ in serum, *P* < 0.05) compared with the CC and TC groups (Figures 3(a)–3(e)); however, higher TG antibody levels were not found (Figure 3(f)). Moreover, thyroid peroxidase (TPO) protein expression was enhanced in the thyroid, but TG protein expression was significantly decreased in the SD group compared with the CC and TC groups (Figures 3(g)–3(i)).

### 3.5. Autophagy Is Decreased in the Thyroid of Sleep-Deprived Rats

**Figure 4:**
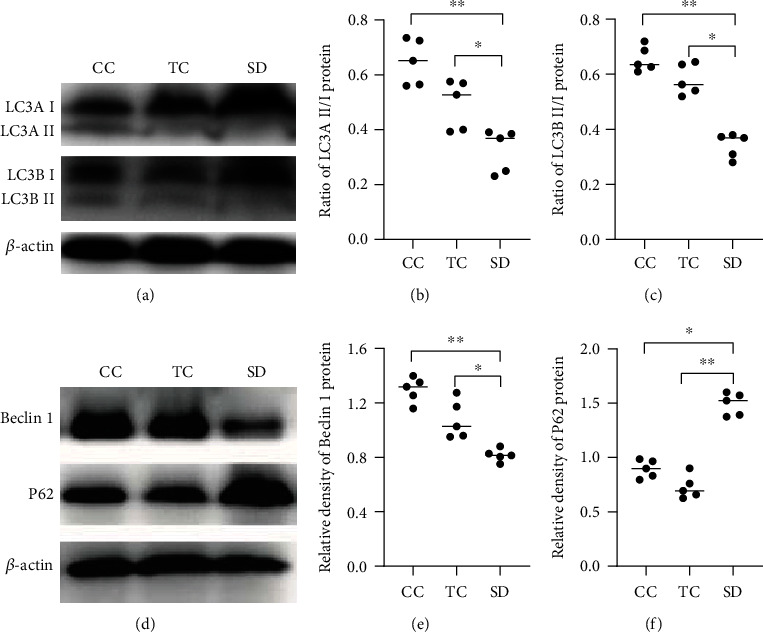
SD inhibited autophagic activity in the thyroid. (a) Expression of LC3. (b) LC3A II/LC3A I ratios. (c) LC3B-II/LC3B-I ratios. (d) The representative expression of Beclin 1 and P62 was measured by western blotting. The relative densities of the bands of (e) Beclin 1 and (f) P62 in each lane were analyzed and normalized to *β*-actin. Data are shown as the median (bar), and scatter plots are shown. ^∗^*P* < 0.05, ^∗∗^*P* < 0.01, compared with rats in the CC or TC group; *n* = 5 in each group.

The levels of the following autophagy-related proteins were determined and analyzed by western blotting to reflect the status of autophagy in the thyroid of sleep-deprived rats: LC3A, LC3B, sequestosome (P62/SQSTM1), and Beclin 1. The LC3A- and LC3B-II/I ratios were significantly lower in the SD group than in the CC and TC groups (Figures 4(a)–4(c)). Additionally, Beclin 1 levels were significantly decreased in the SD group compared with the CC and TC groups, whereas P62 levels were significantly increased in the SD group (Figures 4(d)–4(f)). These findings indicated that sleep deprivation inhibited autophagy in the thyroid.

### 3.6. Sleep Deprivation Promotes Apoptosis in the Rat Thyroid

**Figure 5:**
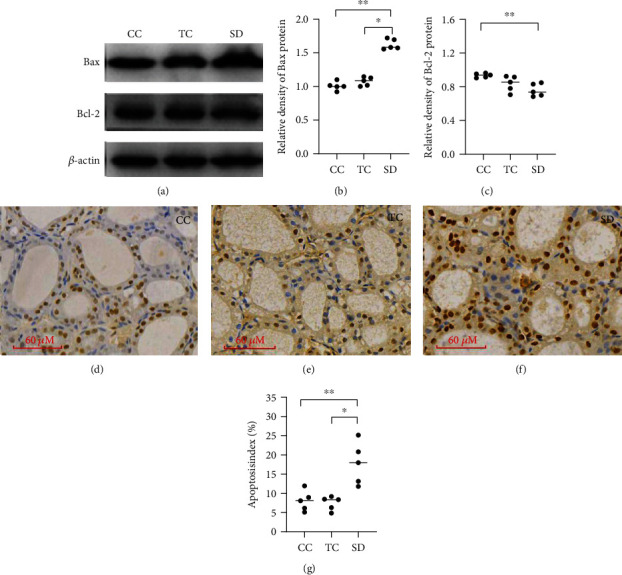
SD affected apoptosis in rat thyroids. (a) Expression of Bax and Bcl-2 was measured by western blotting. The relative densities of the bands of (b) Bax and (c) Bcl-2 in each lane were analyzed and normalized to *β*-actin. Data are shown as the median (bar), and scatter plots are shown. Apoptosis in the thyroid of the CC group (d), TC group (e), and SD group (f). TUNEL staining was used to quantify cell apoptosis, and apoptosis index (TUNEL-positive cells of total cells, %) was analyzed. ^∗^*P* < 0.05, ^∗∗^*P* < 0.01, compared with rats in the CC or TC group; *n* = 5 in each group.

Protein expression of Bcl-2 and Bax was determined as important indicators of apoptosis. Bax protein expression was significantly higher and Bcl-2 protein was significantly lower in the SD group compared with the CC group, but there was no difference in Bcl-2 protein expression between the SD and TC groups. (Figures 5(a)–5(c)). According to a TUNEL assay, more apoptotic cells were found in the thyroid of the SD group than that in the CC group (Figures 5(d)–5(f)).

## 4. Discussion

The metabolic processes of cells, tissues, and organs have a circadian rhythm, which directly affects the body's overall energy metabolism [12]. The thyroid gland plays an important role in energy metabolism. Changes in sleep patterns are undoubtedly associated with changes in thyroid function [13]. Forced sleep deprivation is the main method for studying the impact of insufficient sleep on the human body. Therefore, we established a rat model of partial sleep deprivation for 21 days to observe the effects of sleep deprivation on thyroid function and explore the underlying mechanism.

The experimental results revealed that the thyroid gland exhibited abnormal changes in weight, cell morphology, and various biochemical indicators after sleep deprivation. The weight index of the thyroid was decreased, and vacuolar degeneration was found in thyroid epithelial cells, indicating cell damage, perhaps accompanied by apoptosis, which we confirmed by TUNEL staining of chromatin. TG is considered a special marker of thyroid integrity. When the thyroid is damaged, TG levels decrease, which increases the levels of TSH via a feedback mechanism and further promotes the increase of TPO. TPO is a key enzyme in the synthesis of thyroid hormones, catalyzing the iodination and coupling of TG tyrosine residues accompanied by a complete peroxidation initiation system [14, 15]. The increase of TSH stimulates thyroid cells to secrete T_4_ and T_3_, and the increase of TPO also promotes the synthesis of thyroid hormones. Thyroid hormones are the main regulators of metabolism, growth, and development. The increase of thyroid hormone serum levels directly causes catabolism of proteins, carbohydrates, and lipids, resulting in weight loss.

A key step in thyroid hormone biosynthesis is the iodination of the tyrosyl residues of TG, which depends on the interaction of iodide, TG, H_2_O_2_, and TPO at the apical plasma membrane of thyrocytes [14]. Thyroid hormone synthesis is dependent on the generation of H_2_O_2_. When thyroid function is abnormal, thyroid hormone synthesis is continuously active, and the balance between oxidants and antioxidants can be easily disrupted, resulting in oxidative stress. The levels of oxygen free radicals (reactive oxygen species) increase, resulting in inflammatory cells infiltrating the thyroid and causing tissue damage [16].

Many studies have shown that under conditions of sleep deprivation, the body enters a state of oxidative stress [17]. The thyroid has strong compensatory and antioxidative stress mechanisms, and it contains high concentrations of various antioxidant enzymes. Therefore, the activity of the antioxidant system is enhanced in the thyroid, releasing SOD, GPx, and other antioxidant substances to promote the conversion of reactive oxygen species to harmless substances. However, damage to the body impairs the ability of the antioxidant system to function correctly. As an indirect indicator of the level of oxidative stress, MDA is a metabolite of lipid peroxidation. In the present study, MDA levels were significantly increased and T-AOC was decreased in the SD group, indicating that the rats were in a state of uncompensated oxidative stress.

Reactive oxygen species can play an important role as signaling molecules in the formation of autophagosomes. Oxidative stress has been shown to increase cell survival by triggering autophagy [18], which is the most important way for cells to degrade their own denatured proteins and damaged organelles. Autophagy is related not only to physiological processes such as the development and aging of an organism but also to protective processes against pathological damage [19]. Autophagy is a complex process involving a variety of regulatory proteins, of which Beclin 1 has an important position [20]. Beclin 1 can participate in the formation of autophagosomes through the Beclin 1-PI3KC3 complex and can also promote the maturation of autophagosomes through the Beclin 1-PI3KC3-UVRAG complex. Because of its central role in the formation and maturation of autophagosomes, the Beclin 1 protein can be used to monitor autophagy.

LC3 is an autophagy regulatory protein located in the inner autophagosome membrane [21]. It plays a key role in the elongation and maturation of the autophagosome membrane. When the cell initiates autophagy, the LC3 precursor is processed to form LC3-I in the cytoplasm. LC3-I combines with phosphatidylethanolamine on the surface of the autophagosome membrane to produce LC3-II, where it exerts its function [22]. At the same time, studies have shown that LC3-II levels are directly proportional to the number of autophagic vesicles, so the degree of autophagy is positively correlated with the expression of LC3-II [23]. During autophagy, P62 can be combined with LC3-II and subsequently degraded, causing P62 to be consumed and its expression reduced [24]. Thus, autophagic activity can be determined by measuring the levels of LC3 and P62. In the SD group of the present study, Beclin 1 protein levels and the LC3B(A)-II/I ratio were significantly reduced, whereas P62 protein levels were increased, suggesting that sleep deprivation caused a decrease in thyroid cell autophagy. In the thyroid, the synthesis of H_2_O_2_ potentially is dangerous for thyrocytes, but H_2_O_2_ formed at the surface of thyrocytes will be rapidly used for iodination reactions, and the intracellular H_2_O_2_ is degraded by antioxidant enzymes [24]. As sleep deprivation occurs, the antioxidant capacity is consumed, and the incomplete removal of H_2_O_2_ will also aggravate the oxidative stress of thyroid tissues. So we hypothesize that this might be related to the presence of excessive oxidative stress that makes autophagy unable to compensate for cell damage and inhibits autophagy.

Some studies have pointed out that when autophagy cannot meet the need to clear damaged subcellular structures, excessive reactive oxygen species can inhibit autophagy through the AKT-mTOR-p70S6K pathway, but this damages proteins, membrane lipids, nuclear DNA, and other cellular molecules, ultimately leading to apoptosis [25, 26].

Of the members of the Bcl family, Bcl-2 and Bax have prominent roles in apoptosis; Bcl-2 inhibits apoptosis, whereas Bax promotes it. The relative levels of these two proteins directly determine cell longevity [27]. In addition, reactive oxygen species accumulate in large quantities during oxidative stress and attack the cell membrane system, causing damage to the integrity of the mitochondrial membrane, releasing large amounts of cytochrome c, caspase, and other proteins, and further promoting apoptosis [28]. In the present study, Bax expression was significantly increased in the SD group, which meant more Bax homodimers would be formed, indicating a trend for increased apoptosis in the rat thyroid after sleep deprivation. TUNEL staining of chromatin showed enhanced apoptosis in thyroid cells after sleep deprivation in rats. In other words, sleep deprivation can inhibit autophagy and induce apoptosis in thyroid cells through oxidative stress.

The above experiments showed that the levels of Bcl-2 and Beclin 1, two proteins involved in autophagy and apoptosis [29–31], changed by different degrees. Bcl-2 has been confirmed to interact with Beclin 1, and the degree of binding of Bcl-2 with Beclin 1 determines the occurrence of autophagy [30]. It is reported that Bcl-2 inhibits starvation-induced autophagy in a Beclin 1-dependent manner [32]. The mechanism may be that Bcl-2 inhibits the activation of Beclin 1 during autophagy by binding to it, thus inhibiting autophagy [33]. Our experimental results are consistent with this possibility in the main; compared with TC group, Bcl-2 in the SD group did not decrease significantly. Part of Bcl-2 can combine with Beclin 1 to inhibit autophagy. But the specific mechanism needs to be characterized further. In addition, our experimental design needs to be further improved. We should detect Beclin 1-dependent and non-Beclin 1-dependent autophagy pathways to further clarify the conclusion. We should also carry out downstream detection of autophagy activity, monitoring autophagy, lysosomal fusion, and degradation of intimal vesicles of autophagosomes in the future study.

## 5. Conclusion

In our rat model of sleep deprivation, thyroid function and morphology were changed. The body compensatively maintained metabolism by increasing thyroid activity. The thyroid gland showed apoptosis and follicular hyperplasia, which seem to be related to the imbalance of autophagy and apoptosis of thyroid cells. The complex network of Bcl-2 and Beclin 1, which are important proteins for autophagy and apoptosis, links them together. However, to understand their detailed effects, further studies are necessary to examine a number of different pathways.

## Data Availability

The data used to support the findings of this study are included within this article.
